# Evaluation of indoxacarb and fipronil (s)-methoprene topical spot-on formulations to control flea populations in naturally infested dogs and cats in private residences in Tampa FL. USA

**DOI:** 10.1186/1756-3305-6-366

**Published:** 2013-12-28

**Authors:** Michael W Dryden, Patricia A Payne, Vicki Smith, Monica Chwala, Emery Jones, Jacob Davenport, Gabrielle Fadl, Maria F Martinez-Perez de Zeiders, Kathleen Heaney, Pamela Ford, Fangshi Sun

**Affiliations:** 1 Dept. of Diagnostic Medicine/Pathobiology, Kansas State University, Manhattan, KS 66506, USA; 2 Merck Animal Health, 556 Morris Avenue, Summit, NJ 07901, USA

**Keywords:** Ctenocephalides felis felis, Cat flea, Cats, Dogs, Field study, Fipronil, Flea, Flea control, Indoxacarb, Methoprene

## Abstract

**Background:**

A study was conducted to evaluate and compare the effectiveness of two different spot-on topical flea products to control flea infestations on naturally infested dogs and cats in Tampa, FL USA.

**Methods:**

Thirty-two dogs and 3 cats with natural flea infestations living in 18 homes were treated topically with a 19.53% w/w spot-on formulation of indoxacarb. Another thirty dogs and 2 cats living in 19 different homes were treated topically with either fipronil (9.8% w/w)/(s)-methoprene (8.89% w/w) or fipronil (9.8% w/w)/(s)-methoprene (11.8% w/w), respectively. All products were applied according to label directions by study investigators on day 0 and again between days 28 and 30. Flea populations on pets were assessed using visual area counts and premise flea infestations were assessed using intermittent-light flea traps on days 0, 7, 14, 21, 28–30, 40–45, and 54–60.

**Results:**

A single application of the indoxacarb or fipronil (s)-methoprene formulations reduced flea populations on pets by 97.8% and 85.5%, respectively within 7 days. One month (28–30 days) after treatment the indoxacarb and fipronil (s)-methoprene formulations reduced on-animal flea burdens by 95.0% and 49.5%, respectively. Following two monthly applications of either the indoxacarb or fipronil (s)-methoprene formulations, pet flea burdens were reduced by 99.1% and 54.8%, respectively, by days 54 – 60. At the end of the two month study, 77.1% and 15.6% of the dogs and cats in the indoxacarb and fipronil (s)-methoprene treatment groups, respectively were flea free. Flea numbers in the indoor-premises were markedly reduced in both treatment groups by days 54–60, with 97.7% and 84.6% reductions in intermittent-light flea trap counts in the indoxacarb and fipronil (s)-methoprene treatment groups, respectively.

**Conclusions:**

This in-home investigation conducted during the summer of 2013 in subtropical Tampa, FL, is the first published U.S field investigation of the indoxacarb topical formulation. The indoxacarb formulation was able to effectively control flea populations in heavily flea infested pets and homes. The efficacy achieved by the fipronil (s)-methoprene formulation against flea infestations on these pets was lower than in previous investigations using the same study design.

## Background

Fleas are likely the most common ectoparasite infesting dogs and cats. Elimination and prevention of flea infestations are critical to maintaining good health because fleas are not just irritating, but clinically relevant, causing anemia, being responsible for allergic dermatitis, vectoring various bacterial pathogens, and serving as the intermediate host for filarid and cestode parasites in dogs and cats [1].

Previous studies conducted in the United States, Europe and Australia have demonstrated that dinotefuran-pyriproxyfen, fipronil (±, (s)-methoprene) imidacloprid, imidacloprid-flumethrin, lufenuron (+pyrethrin spray or + nitenpyram tablets) selamectin, and spinosad were effective in markedly reducing or eliminating natural flea infestations on dogs and cats and in private residences without the need for treatment of the premises [2-10]. Flea infestations were eliminated or dramatically reduced in these studies by controlling flea reproduction by either killing most newly acquired fleas prior to initiation of egg laying and/or rendering the vast majority of deposited eggs non-viable. Since the introduction of the modern topical and systemic products in the mid-1990s it appears that flea control on pets and in their owners’ homes succeeds or fails based upon a product or combination of products’ ability to suppress flea reproduction [6,7].

The objective of this current study was to evaluate the performance of a new indoxacarb containing topical spot-on formulation to eliminate natural flea infestations on dogs and cats in Tampa, FL USA. Indoxacarb is considered a pro-insecticide that is bioactivated by esterase and amidase enzymes within the insect to a more active metabolite [11,12]. A previous laboratory study conducted at Kansas State University demonstrated that a single topical spot-on formulation of indoxacarb applied to cats provided ≥99.6% efficacy against flea infestations for 6 weeks [13]. This treatment also markedly reduced or eliminated egg production for 45 days and reduced the viability of the few eggs that were produced for up to 38 days following a single treatment [13]. These laboratory data indicated that the formulation might be able to effectively control flea populations in naturally flea infested pets and their homes. The positive reference control chosen for this study was fipronil (s)-methoprene. Both fipronil only and fipronil (s)-methoprene topical spot-ons have been used previously in our studies in Tampa with good results [4,6,7].

The effectiveness of the treatments was assessed using both premises and on-animal flea population estimating techniques. The study design used in this investigation eliminated client and veterinarian perceptions of performance and reduced client compliance problems because investigators administered products to all animals.

## Methods

### Home and pet study inclusion criteria

Through referrals from Sunshine Animal Hospital, Tampa, FL and advertisements on CRAIGSLIST®, 42 private residences were selected for inclusion in the study from May 21 - June 01, 2013.

Homes were selected based on the following criteria: 1) a minimum of five fleas observed in area flea counts on at least one dog at the residence; 2) a minimum of five fleas collected in a 16 – 24 hour period in two intermittent light flea traps; 3) one to five healthy, non-fractious dogs or cats at the residence; 4) qualifying pets must spend the majority of their time in the indoor premises; 5) homeowner’s willingness to participate in the 2 month study; 6) owners agreeing not to use any other topical or premise flea control products during the study; 7) no dog or cat in the household can be pregnant or nursing; 8) completion of a questionnaire concerning pet habits, visiting pets, previous flea treatments and personal observations around their residence concerning wildlife and feral cats and 9) owners willingness to sign an informed consent form.

All dogs and cats were client-owned, resided in a private residence and were handled and treated in compliance with Kansas State Institutional Animal Care and Use Committee (IACUC #3281) approval.

### Treatment groups

Homes and pets meeting these criteria were placed into 1 of 2 treatment groups. Home entry numbers (1–42) were each assigned a random number by Excel (Excel 2010) and blocked into groups of 2. The highest random number within each block was assigned to group 1 and lowest to group 2.

Dogs and cats in treatment group 1 were treated topically with indoxacarb (19.53% w/w; ACTIVYL®, Merck Animal Health).

Dogs in group 2 were treated topically with fipronil (9.8% w/w)–(s)-methoprene (8.8% w/w) spot-on (Frontline® Plus, Merial) and cats in group 2 were treated topically with fipronil (9.8% w/w)–(s)-methoprene (11.8% w/w) (Frontline®Plus, Merial).

All pets were weighed prior to each treatment and products were administered using the commercial product applicator. Treatment was administered by parting the hair with the tip of the applicator and the entire contents were applied directly to the skin in one or more spots as indicated by the label. While only pets meeting the inclusion criteria were included in the study for data collection, all dogs and cats living at a residence or reported to investigators as a visiting pet, were administered appropriate treatments. Dogs and cats were treated once on day 0 and once between days 28 to 30. All treatments were administered by members of the Kansas State University (K-State) Flea Team who were not blinded to treatment groups. No other topical or in-door premises flea treatments were used during the 60 days of the study. There were no restrictions on the animals regarding exposure to rain, swimming, or movement outdoors. Owners were asked not to bathe their pets more than twice a month.

### Flea population assessment

The numbers of adult fleas present in the indoor premises were assessed using intermittent light traps [3-7,14,15]. One trap was placed in each of two rooms for 16 to 24-hours. Rooms were selected based on where the pet(s) spent most of the time or where owners had observed fleas. Once rooms were selected, the traps were returned to the same rooms in the same location at every counting period. Fleas collected on the adhesive pads of the traps were enumerated and identified to species.

The flea population on each pet was assessed using a visual area count methodology [3-7,16]. Area counts were performed at five locations on each animal; dorsal midline, tail head, left lateral, right lateral, and inguinal region. Area counts were limited to one minute per location and conducted by parting the hair against the lay using both hands until the area was covered. Maximum number of fleas per zone was capped at 50; therefore the maximum total area flea counts for a pet was 250. Pet and premises flea counts were conducted on days 0, 7, 14, 21, 28–30, 40–45, and 54–60. Personnel conducting pet and premises flea counts were not blinded to treatment groups.

### Data analysis

The flea counts in home and flea counts on animal data were log transformed for analysis. A value of one was added to all observations to make log transformation applicable. Data were analyzed for each count day separately. The log transformed data were analyzed by a mixed linear model including fixed effect treatment and random effects home and home* treatment.

Kenward-Roger correction was used to determine the denominator degrees of freedom. Least squares means were used for treatment comparisons and were back transformed to obtain the estimates for the geometric mean at each week.

The numbers of flea free animals were compared using Fisher’s exact test.

The null hypothesis is that there is no significant difference between the two treatment groups. Two tailed tests were used for the comparison. Statistical significance was declared when P ≤ 0.05. The primary software was SAS version 9.3.

Percentage efficacy achieved by the flea products was calculated by the following formula: {(Day 0 Geometric Mean Flea Counts [pet or trap] - Day x Geometric Mean Flea Counts [pet or trap])/(Day 0 Geometric Mean Flea Counts [pet or trap])} multiplied by 100.

## Results

Forty-two homes were originally enrolled in the study. However, three households in the indoxacarb study group and two households in the fipronil (s)-methoprene group did not complete the study and therefore data from those households were not included. In one of the households in the indoxacarb treatment group, the single enrolled pet was not at the home to be counted on days 21 or 28 – 30; in another household in that treatment group one of the dogs displayed extreme aggressiveness and mauled another dog in the home; and, in the third home, the owner moved to another residence one week into the study. In the fipronil (s)-methoprene treatment group, the only dog on study in one home displayed extreme aggressiveness one week into the study, and in the other home the only dog on study died of a gastric torsion one week into the study. In none of these cases was product application deemed a cause of homes and the pets being removed from the study.

In the 18 homes that completed the study where pets were treated with indoxacarb, there were 32 dogs (avg. 15.4 kg; range 2.1 – 43.0 kg) and 3 cats (avg. 4.4 kg; range 3.1 – 6.3 kg) officially enrolled. On day 0 the dogs received a mean topical dose of 22.8 mg/kg (range 15.3 – 47.6 mg/kg) indoxacarb. The cats received a mean topical dose of 30.1 mg/kg (range 26.1 – 32.2 mg/kg) indoxacarb. There were an additional 8 dogs and 8 cats in these homes that did not qualify for the study because they: had an insufficient numbers of fleas (<5) on day 0, resided permanently outdoors, were brought into the home after the enrollment date, or could not be safely handled by flea team members to conduct flea counts. Therefore, there were a total of 40 dogs and 11 cats resident in the 18 homes that were treated with indoxacarb.

In the 19 homes that completed the study where pets were treated with fipronil (s)-methoprene there were 30 dogs (avg. 18.7 kg; range 2.1 – 49.6 kg) and 2 cats (avg. 4.6 kg; range 1.6 – 7.7 kg) officially enrolled in the study. On day 0 the dogs received a mean dose of 12.9 mg/kg (range 7.2 – 32.8 mg/kg) fipronil and 11.6 mg/kg (range 6.4 – 29.5 mg/kg) (s-)-methoprene. The cats in these homes received a mean topical dose of 19.0 mg/kg (range 6.5 – 31.4 mg/kg) fipronil and 22.8 mg/kg (range 7.8 – 37.7 mg/kg) (s)-methoprene. There were an additional 8 dogs and 8 cats in these homes that did not qualify for the study because of the same factors listed previously. Therefore, there were a total of 38 dogs and 10 cats resident in the 19 homes that were treated with fipronil (s)-methoprene.

On day 0, pets in the indoxacarb treatment group had a geometric mean of 51.0 (range 6 – 250) fleas observed in area counts (Table 1). Pets in the fipronil (s)-methoprene treatment group had a geometric mean of 28.1 (range 5 – 250) fleas observed in area counts on day 0. There was one household with 5 dogs in the indoxacarb treatment group that clearly raised the overall geometric mean of that treatment group. Four of the dogs met the upper limit for area counts at 250 fleas per dog and the other dog had 208 fleas in the area counts. If the area flea counts from the dogs in that household were eliminated, the geometric mean of the indoxacarb treatment group would have been 39.2.

**Table 1:** Geometric mean and percent control of on-animal flea counts in naturally infested homes when dogs and cats were treated with indoxacarb or fipronil (s)-methoprene based topical spot-on formulations

**Treatment group**	**# dogs/cats**		**Days post-treatment**^**1**^						
			**Day 0**	**Day 7**	**Day 14**	**Day 21**	**Day 28 – 30**	**Day 40 - 45**	**Day 54 - 60**
Indoxacarb^2^	32/3	Geomean^4^	51.0^a^	1.1^a^	0.6^a^	1.3^a^	2.6^a^	0.3^a^	0.5^a^
		% control^5^		97.8	98.8	97.4	95.0	99.4	99.1
		% (#) pets with no fleas	0.0	48.6	71.4	40.0	20.0	71.4	77.1
			(0/35)	(17/35)	(25/35)	(14/35)	(7/35)	(25/35)	(27/35)
Fipronil (s)-methoprene^3^	30/2	Geomean	28.1^a^	4.1^b^	6.4^b^	13.6^b^	14.2^b^	8.0^b^	12.7^b^
		% control		85.5	77.3	51.5	49.5	71.6	54.8
		% (#) pets with no fleas	0.0	15.6	15.6	9.4	6.3	21.9	15.6
			(0/32)	(5/32)	(5/32)	(3/32)	(2/32)	(7/32)	(5/32)

^1^Dogs and cats treated on day 0 and again between days 28 – 30.

^2^Dogs and cats were treated topically with indoxacarb (19.53% w/w; ACTIVYL® Merck Animal Health) according to label directions.

^3^Dogs were treated topically with fipronil (9.8% w/w)–(s)-methoprene (8.8% w/w) spot-on (Frontline®Plus; Merial) and cats were treated topically with fipronil (9.8% w/w)–(s)-methoprene (11.8% w/w) (Frontline®Plus; Merial) according to label directions.

^4^Geometric mean numbers of fleas in visual area counts on pets.

^5^{(Day 0 geometric mean animal area flea counts – day x geometric mean animal area flea counts)/day 0 geometric mean animal area flea counts)} x 100

^a,b^Geometric means in a column with unlike letter superscripts are significantly different (P values ≤0.0079).

Within 7 days of application of indoxacarb or fipronil (s)-methoprene the flea counts were reduced by 97.8% and 85.5%, respectively (Table 1). By days 28–30 the flea counts in the indoxacarb treatment group were reduced by 95.0% and following reapplication at one month, total pet flea burden was reduced by 99.1% by days 54–60 (Table 1). Fipronil (s)-methoprene did not reduce flea populations as effectively, with 49.5% control on day 28–30 and only 54.8% reduction by days 54 – 60 (P ≤ 0.0079) (Table 1).

In the indoxacarb treatment group there were significantly more flea free pets by the end of the study than in the fipronil (s)-methoprene treatment group (p < 0.0001) (Table 1). In the indoxacarb treatment group 77.1% (27/35) of pets were completely flea free by days 54–60. Whereas only 15.6% (5/32) of the pets in the fipronil (s)-methoprene treatment group were flea free by the end of the study (Table 1).

The client interviews conducted before homes were entered into the study and again upon completion of the study provide some interesting insights into pet habits and outdoor presence of potential flea hosts. Owners reported traveling to other homes with their pet(s) and having other pets visit their homes, sometimes for several days. In the indoxacarb and fipronil (s)-methoprene treatment groups 38.9% (7/18) and 42.1% (8/19) of the homes had a visitor dog during the study, respectively. In these cases the investigators were notified and visitor pets were treated. In the indoxacarb and fipronil (s)-methoprene treatment groups, dogs from 27.8% (5/18) and 21.1% (4/19) of the homes visited another home, however, flea treatment status of pets resident in these other homes could not be reliably determined.

Potential flea hosts were observed in the immediate outdoor premises of the homes of pet owners in both treatment groups. Pet owners in the indoxacarb treatment group said they had seen opossums (77.8%; 14/18), raccoons (61.1%; 11/18) or feral cats (100.0%; 18/18) in their yards. The fipronil (s)-methoprene treatment group was similar with 94.7% (18/19) of pet owners indicating they had seen opossums (68.4%; 13/19), raccoons (36.8%; 7/19) or feral cats (89.5%; 17/19) in their yards.

During the entire 2 month study, 5,418 fleas were trapped in the 37 residences using intermittent light traps and all were identified as *Ctenocephalides felis felis*, the cat flea. On day 0, the traps collected a geometric mean of 29.5 (range 5 – 390) and 25.5 (range 5 – 457) fleas in homes in the indoxacarb and fipronil (s)-methoprene treatment groups, respectively (Table 2). Reductions in emerging flea populations were 87.9% and 97.7% by days 28–30 and 54 – 60, respectively in the homes where pets were treated with indoxacarb (Table 2). Reductions in emerging flea populations were 60.3% and 84.6% by days 28–30 and 54 – 60, respectively in the homes where pets were treated with fipronil (s)-methoprene (Table 2). The geometric mean number of fleas was significantly lower on post-treatment days 40–45 and 54–60 in the homes where pets were treated with indoxacarb (p ≤ 0.0054) (Table 2).

**Table 2:** Geometric mean and percent control of fleas recovered in premises flea traps in naturally infested homes when dogs and cats were treated with indoxacarb or fipronil (s)-methoprene based topical spot-on formulations

**Treatment group**	**# homes completing study**		**Days post-treatment**^**1**^						
			**Day 0**	**Day 7**	**Day 14**	**Day 21**	**Day 28 – 30**	**Day 40 - 45**	**Day 54 – 60**
Indoxacarb^2^	18	Geomean^4^	29.4^a^	8.1^a^	5.2^a^	3.8^a^	3.5^a^	0.9^a^	0.7^a^
		% control^5^		72.4	82.4	87.2	87.9	97.0	97.7
Fipronil (s)-methoprene^3^	19	Geomean	25.5^a^	9.5^a^	7.5^a^	7.0^a^	10.1^a^	5.1^b^	3.9^b^
		% control		62.6	70.4	72.4	60.3	79.9	84.6

^1^Dogs and cats treated on day 0 and again between days 28 – 30.

^2^Dogs and cats were treated topically with indoxacarb (19.53% w/w; ACTIVYL® Merck Animal Health) accordingly to label directions.

^3^Dogs were treated topically with fipronil (9.8% w/w)–(s)-methoprene (8.8% w/w) spot-on (Frontline®Plus; Merial) and cats were treated topically with fipronil (9.8% w/w)–(s)-methoprene (11.8% w/w) (Frontline®Plus; Merial) according to label directions.

^4^Geometric mean numbers of fleas recovered in two intermittent light flea traps.

^5^{(Day 0 geometric mean trap flea counts – day x geometric mean trap flea counts)/day 0 geometric mean trap flea counts)} x 100

^a,b^Geometric means in a column with unlike letter superscripts are significantly different (P values ≤0.0054).

During the 2 month study, three households in the indoxacarb treatment group reported adverse health events in four dogs. In one household, the owner reported that the dog slept more the night after the first treatment and would not eat. Upon examination of the dog the next day, the dog was active, alert and ate when offered food. In another home the owner reported the dog “whined” at night following the first treatment, but the dog was reported as normal the next day. It is unknown if either of these reports were product related. In another home both dogs on study vomited during the night following the first treatment. Neither dog vomited following the second treatment at one month. The vomiting episodes in these two dogs may or may not have been product related. There were no adverse health events reported in homes where pets were treated with fipronil (s)-methoprene.

## Discussion

The indoxacarb topical spot-on formulation used in this investigation provided excellent flea control achieving >99% reductions in pet area counts after two monthly applications. The residual activity of this formulation was remarkable given the continual reinfestation pressure from the heavily infested premises. The fipronil (s)-methoprene topical spot-on formulation did not eliminate fleas as effectively as the indoxacarb formulation. Following two monthly applications of fipronil (s)-methoprene the reduction in pet area flea counts was only 54.8% by the end of the two month study.

The difference in efficacy between the two formulations in this study was further demonstrated when looking at the percent of pets that had no fleas at the end of the study. Of the pets treated with indoxacarb, 77.1% were flea free by the end of the 2 month study, whereas only 15.6% of the pets in the fipronil (s)-methoprene treatment group were flea free by the end of the study.

The level of efficacy observed in this study for the fipronil based topical spot-on was less than that observed previously in Tampa using the same study design. In 1997 the percent reduction in pet flea counts after two monthly applications of a fipronil only based topical spot on was 99.2% by day 60 [4]. In 2009 and 2010 the same fipronil (s)-methoprene topical spot-on formulation that was used in the current study was evaluated and the reduction in pet flea counts by day 60 were 87.5% and 95.5%, respectively [6,7]. It is unknown why two treatments with the fipronil (s)-methoprene combination did not effectively eliminate flea infestations on pets in this current study. The reduced efficacy of the fipronil formulation could be the result of resistance, innately tolerant flea strains or potentially other factors as yet unknown.

The area count technique used in this and previous in-home investigations has been shown to detect an average of 23.5% of the total pet flea burden [16]. Therefore, average pretreatment total body flea burdens of pets in the indoxacarb and fipronil (s)-methoprene treatment groups based on geometric means area counts of 51.0 and 28.1 can be estimated to be approximately 217 and 119 for pets in this study, respectively. These are remarkably high flea burdens and it is particularly interesting that the geometric mean area flea counts observed in this study for pets in the indoxacarb treatment group were higher than in any previously published studies these investigators have conducted in Tampa. Prior to this study, the geometric mean pet counts recorded in 2010 had been the highest at 28.5 [7]. It is unknown why pet flea counts in the 2010 and 2013 studies were higher than in previous years in Tampa. Factors that might affect flea numbers include: annual differences in temperature or humidity, treatment history, number of pets in a household, number of indoor-outdoor pets, household air conditioning, and outdoor flea reservoir populations.

It is notable that in both treatment groups in the current study there were dogs whose flea infestation levels met or exceeded our maximum pet area count of 250 fleas. Previous studies have indicated that flea infestation levels above 50 in a specific region cannot be reliably counted. Based on the area count methodology employed (23.5% of the total pet flea burden) and previous investigations conducted by these authors, those pets where all 5 body areas examined met or exceeded 50 fleas are actually infested with over 1,000 fleas.

It is important to note that while the reduction in pet flea burdens in the indoxacarb treatment group was ≥ 95.0% during the first month it was not uncommon to observe at least some fleas on animals during this post treatment period (Table 1). While a slight drop in efficacy was observed by the end of the first month, as would be expected for any residual insecticide, the better indicator of the reduction in speed of kill of a product may be more readily observed in the percent of flea free dogs. From day 14 post-treatment to the end of the first month, the percent of dogs with 0 fleas dropped from 71.4% to 20.0%. These data represent the observations that pet owners are likely to make following treatment of their pets with any insecticide formulation. Pets in this study were under unceasing reinfestation pressure from fleas emerging continuously in their homes. As speed of kill naturally wanes throughout the month following treatment, pet owners will observe more fleas on their pets. By the end of the two month study, even though efficacy against on-animal flea burdens on indoxacarb treated pets was 99.1%, the number of completely flea free pets was 77.1%.

Previous in-home studies conducted in Tampa have also demonstrated that percent reductions in total pet flea burden are often less than 100% even through 90 days of treatment [3-7]. In the 2010 study where pets were treated with either a dinotefuran-pyriproxyfen or fipronil (s)-methoprene topical spot-on formulation, only 60.0% and 55.6% of pets were completely flea free by days 54 – 60 [7].

The residual efficacy of the fipronil (s)-methoprene formulation was significantly less against the on-animal flea burdens in this study than the indoxacarb formulation, and therefore an even more dramatic drop off in efficacy was observed throughout the month following application. Efficacy of the fipronil (s)-methoprene formulation fell from 85.5% on day 7 to 49.5% on day 30 post-treatment. Correspondingly, the percent of pets with 0 fleas fell from 15.6% on day 7 to 6.3% on day 30.

These studies over several years document that it should be expected to see fleas on some treated pets for 2 to 3 months following treatment. This is particularly evident in areas with naturally high levels of flea infestations. Data on the percent of flea free pets post-treatment in a multicentric study conducted in Europe are similarly informative [2]. In that study, data from Spain, a country with a high initial flea infestation rate, demonstrated that 70.3-72.7% of dogs and cats were flea infested on day 0. Whereas after two monthly applications of fipronil (s)-methoprene, 33.3%-34.6% of dogs and cats, still had fleas.

The intermittent-light flea traps used in these in-home investigations provide an estimate of development and flea emergence in a home. Following two monthly applications of indoxacarb or fipronil (s)-methoprene topical spot-on formulations to the pets, premises flea counts were reduced by 97.7% and 84.6% respectively. A previous laboratory study demonstrated that a single topical spot-on treatment of indoxacarb to cats eliminated or dramatically reduced egg production and reduced the viability of the few eggs that were produced for up to 6 weeks after treatment [13]. Given the effect of indoxacarb on adult fleas, flea egg production and egg viability, the 97.7% reduction in emerging flea populations observed in this in-home investigation indicates that indoxacarb greatly reduced viable flea reproduction under these tough field conditions.

The efficacy of indoxacarb against fleas infesting pets and fleas emerging in these homes was similar, but that was not observed for the fipronil (s)-methoprene formulation. The flea population reductions in premises at the end of the study in homes with fipronil (s)-methoprene treated pets were almost 30 percent higher than reductions in on-animal flea counts. While it is difficult to draw any definite conclusions, these authors speculate that the ovicidal activity of the (s)-methoprene on eggs laid by fleas on treated pets may account for this observation.

A challenge for many pet owners and their pets is the flea reinfestation pressure from flea infested urban wildlife as well as dogs and cats. In North America, feral cats and urban wildlife such as opossums and raccoons can be infested with *Ctenocephalides felis*, which can deposit flea eggs and contaminate protected outdoor premises such as crawlspaces, decks, and under bushes [1]. Given the potential for reinfestation, it should be of concern that 94.6% of pet owners in this study had seen feral cats and 73.0% also reported observing either opossums or raccoons in their yards. Opossums and raccoons are naturally nocturnal; therefore the true number of owners’ yards frequented by wildlife is likely much higher. Given that such a large percentage of owners’ yards are frequented by potentially flea infested animals, it is understandable that dogs and cats in this subtropical climate face substantial and continuous flea infestation pressure. These pets need to be on year-round and life-long flea control, otherwise re-infestation of the home is almost certain [17].

Another important aspect of this study is that investigators entered homes repeatedly over the two month study. This afforded investigators the opportunity to have repeated interaction with pet owners to ascertain whether other pets were brought into the home and whether enrolled pets traveled to other homes with pets. On average 40.5% of homes had dogs visit during the study. Clearly this could be problematic if those dogs were infested with fleas and then deposited eggs into the home. In this study we attempted to account for such occurrences by making sure that visitor dogs were treated with group appropriate treatments. However, there could still have been other visitor pets that were not identified during the study. Veterinary practitioners need to consider that these visitor pets could contribute to perceived failure of a prescribed flea control program.

## Conclusions

This in-home investigation conducted during the summer of 2013 in subtropical Tampa, FL is the first published US field investigation of a novel indoxacarb based topical formulation. In this investigation the indoxacarb formulation outperformed a fipronil (s)-methoprene formulation and was able to effectively control flea populations in homes and on pets even though these pets had the largest natural flea burdens these investigators had ever recorded in Tampa FL. The efficacy achieved by the fipronil (s)-methoprene formulation against flea infestations on these pets was lower than in previous investigations in this area using the same study design.

This study was conducted without a placebo control group. While the use of a non-treated group might have provided a better evaluation of the performance of the two treatment regimens, it is the opinion of these authors that the massive flea infestations commonly encountered in Tampa, FL preclude the use of a non-treated group. Withholding treatment would be detrimental to the health and welfare of the dogs, cats and potentially even to humans in a household.

## Competing interests

MWD has been sponsored at lectures by Merck Animal Health and Merial Animal Health, manufacturers of ACTIVYL® and Frontline® Plus that were evaluated in these investigations. KH, PF and FS are currently employed by Merck Animal Health.

## Authors’ contributions

MWD was primary author of study design, served as primary study investigator and drafted the manuscript. VS and JD coordinated and supervised data collection and entry and revision of manuscript. MC, EJ, JD, GF and MM-PZ were responsible for animal handling and collection of data and data entry. KH assisted in design of study, monitoring of study and manuscript revision. PF assisted in monitoring of study and manuscript revision. FS conducted the statistical analysis of the data. All authors reviewed and approved the final manuscript.

## References

[B1] Blagburn BL Dryden MW Biology, treatment and control of flea and tick infestations Vet Clin N Am 2009 6 6 1173 1200 10.1016/j.cvsm.2009.07.001 19932369

[B2] Beugnet F Franc M Results of a European multicentric field efficacy study of fipronil-(S) methoprene combination on flea infestation of dogs and cats during 2009 summer Parasite 2010 6 4 337 342 10.1051/parasite/2010174337 21275240

[B3] Dryden MW Perez HR Ulitchny DM Control of flea populations on naturally infested dogs and cats and in private residences with either topical imidacloprid spot application or the combination of oral lufenuron and pyrethrin spray Am J Vet Med Assoc 1999 6 1 36 39

[B4] Dryden MM Magid-Denenberg T Bunch S Control of fleas on naturally infested dogs and cats and in private residences with topical spot applications of fipronil or imidacloprid Vet Parasitol 2000 6 69 75 10.1016/S0304-4017(00)00318-6 11027862

[B5] Dryden M Maggid-Denenberg T Bunch S Schenker R Control of fleas on dogs and cats and in private residences with the combination of oral lufenuron and nitenpyram Vet Therapeutics 2001 6 208 214 19746663

[B6] Dryden M Carithers D McBride A Riggs B Smith L Davenport J Smith V Payne P Gross S A comparison of flea control measurement methods for tracking flea populations in highly infested private residences in Tampa FL, following topical treatment of pets with FRONTLINE® Plus (fipronil/(S)-methoprene) Intern J Appl Res Vet Med 2011 6 4 356 567

[B7] Dryden MW Payne PA Smith V Riggs B Davenport J Kobuszewski D Efficacy of dinotefuran–pyriproxyfen, dinotefuran–pyriproxyfen–permethrin and fipronil–(S)-methoprene topical spot-on formulations to control flea populations in naturally infested pets and private residences in Tampa, FL Vet Parasitol 2011 6 281 286 10.1016/j.vetpar.2011.05.054 21705147

[B8] Miller PF Peters BA Hort CA Comparison of lufenuron and nitenpyram versus imidacloprid for integrated flea control Vet Ther 2001 6 4 285 292 19746650

[B9] Robertson-Plouch C Baker KA Hozak RR Zimmermann AG Parks SC Herr G Hart LM Jay J Hutchens DE Snyder DE Clinical field study of the safety and efficacy of spinosad chewable tablets for controlling fleas on dogs Vet Ther 2008 6 1 26 36 18415944

[B10] Stanneck D Rass J Radeloff I Kruedewagen E Le Sueur C Hellmann K Krieger K Evaluation of the long-term efficacy and safety of an imidacloprid 10%/flumethrin 4.5% polymer matrix collar (Seresto®) in dogs and cats naturally infested with fleas and/or ticks in multicentre clinical field studies in Europe Parasit Vectors 2012 6 66 10.1186/1756-3305-5-66 22463745 PMC3353155

[B11] Wing KD Andaloro JT McCann SF Salgado VL Gilbert LI, Gill SS Indoxacarb and the sodium channel blocker insecticides: chemistry, physiology and biology in insects Insect control biological and synthetic agents 2010 London: Elsevier, B.V 35 57

[B12] Wing KD Sacher M Kagaya Y Tsurubuchi Y Mulderig L Connair M Schnee M Bioactivation and mode of action of the oxadiazine indoxacarb in insects Crop Prot 2000 6 537 545 10.1016/S0261-2194(00)00070-3

[B13] Dryden MW Payne PA Smith V Heaney K Sun F Efficacy of indoxacarb applied to cats against the adult cat flea, Ctenocephalides felis, flea eggs and adult flea emergence Parasit Vectors 2013 6 126 10.1186/1756-3305-6-126 23642104 PMC3651268

[B14] Dryden M Broce A Development of a flea trap for collecting newly emerged Ctenocephalides felis (Siphonaptera: Pulicidae) in homes J Med Entomol 1993 6 901 906 8254638 10.1093/jmedent/30.5.901

[B15] Müller GC Dryden MW Revay EE Kravchenko VD Broce AC Hampton K Junnila A Schlein Y Understanding attraction stimuli of Ctenocephalides felis for non-chemical control methods Med Vet Entomol 2011 6 4 413 420 10.1111/j.1365-2915.2011.00960.x 21787369

[B16] Dryden M Boyer J Smith V Techniques for estimating on animal populations of Ctenocephalides felis (Siphonaptera: Pulicidae) J Med Entomol 1994 6 631 624 7932613 10.1093/jmedent/31.4.631

[B17] Dryden MW How you and your clients can win the flea control battle Vet Med 2009 http://veterinarymedicine.dvm360.com/vetmed/ArticleStandard/Article/detail/585264

